# *Entropy* 2021 Best Paper Award

**DOI:** 10.3390/e23070865

**Published:** 2021-07-06

**Authors:** Entropy Editorial Office

**Affiliations:** MDPI, St. Alban-Anlage 66, 4052 Basel, Switzerland; entropy@mdpi.com

On behalf of the Editor-in-Chief, Prof. Dr. Kevin H. Knuth, we are pleased to announce the Entropy Best Paper Awards for 2021.

Papers published in 2019 were preselected by the Entropy Editorial Office based on the number of citations and downloads from the website. The winner nominations were made by a selection committee, which was chaired by the Editor-in-Chief and supported by thirteen Editorial Board Members. The four top-voted papers, in no particular order, have won the 2021 Entropy Best Paper Award:

**Classical (Local and Contextual) Probability Model for Bohm–Bell Type Experiments: No-Signaling as Independence of Random Variables** **[1]**

**Andrei Khrennikov and Alexander Alodjants**

***Entropy* 2019, *21(2),* 157;** https://www.mdpi.com/1099-4300/21/2/157

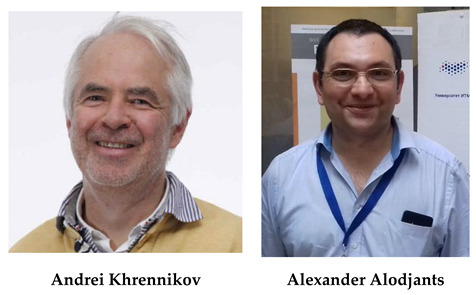



The problem of interrelation between classical and quantum probability is fundamental for understanding links between classical and quantum physics. Our aim is to show that, in spite of the common opinion, correlations of observables *A*1,*A*2 and *B*1,*B*2 involved in the experiments of the Bohm–Bell-type can be expressed as correlations of classical random variables *a*1,*a*2 and *b*1,*b*2. The crucial point is that correlations ⟨*Ai*,*Bj*⟩ should be treated as conditional on the selection of the pairs (*i*,*j*). The setting selection procedure is based on two random generators *R_A_* and *R_B_*. They are also considered as observables, supplementary to the “basic observables” *A*_1_,*A*_2_ and *B*_1_,*B*_2_. These observables are absent in the standard description, e.g., in the scheme for the derivation of the CHSH-inequality. We represent them by classical random variables *r_a_* and *r_b_*. 

Constructing the classical probability representation for quantum correlations demystifies quantum mechanics. Instead of quantum nonlocality (spooky action at a distance) or rejection of realism, we proceed within the standard classical conditional probability framework (local and not less realistic than the framework of classical statistical mechanics). The exotic properties of Bohm–Bell correlations can be explained by ignorance of the important source of randomness—the selection of orientations of the polarization beam splitter. Although this selection is widely discussed, in particular, in relation to the experimenter’s free will, its mathematical counterpart is absent the derivation of the Bell-type inequalities. This problem can be called the “setting selection loophole”. By closing this loophole, we showed that the Bell-type inequalities are violated only by conditional correlations and unconditional correlations satisfy them. However, Bell’s derivation is based on unconditional correlations. In short, an unconditionally derived result is commonly applied to conditional correlations (theoretical and experimental). We also apply the conditional correlation approach to characterize (no-)signaling in the classical probabilistic framework. Consideration of the Bohm–Bell experimental scheme in the presence of signaling is important for applications outside quantum physics, e.g., in psychology and social science.

**Nonlinear Information Bottleneck** **[2]**

**Artemy Kolchinsky, Brendan D. Tracey and David H. Wolpert**

***Entropy* 2019, *21(12),* 1181;** https://www.mdpi.com/1099-4300/21/12/1181

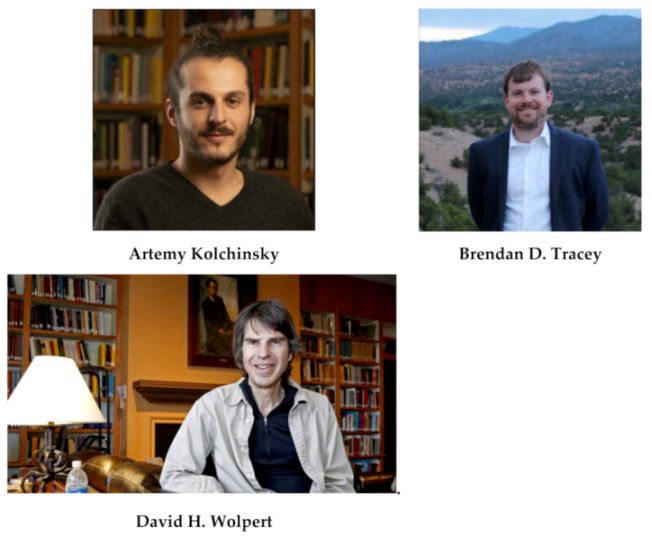



An SFI-authored paper on “Nonlinear Information Bottleneck” is one of four papers to win the 2021 *Entropy* Best Paper Awards, having competed against every other article published by the journal in 2019.

In the paper, SFI Program Postdoctoral Fellow Artemy Kolchinsky, then-Postdoctoral Fellow Brendan Tracey (now at DeepMind), and Professor David Wolpert explored what would happen if artificial neural networks were explicitly trained to discard useless information, as well as making use of a novel estimator developed by Kolchinsky and Tracey.

Said Kolchinsky in 2019, “It may be that deep learning networks succeed because of what they learn to ignore, not just what they learn to predict. So we ask: what happens if we explicitly encourage a network to forget irrelevant information?”

Tracey explained that the motivation for the paper was to “make predictions using data from a bandwidth-limited environment,” such as “a satellite in space, or a remote weather station in Antarctica. You can’t send back all of the data you collect, so which pieces of data are the right data to transmit?”

**Dynamic Maximum Entropy Reduction** **[3]**

**Václav Klika, Michal Pavelka, Petr Vágner and Miroslav Grmela**

***Entropy* 2019, *21(7),* 715;** https://www.mdpi.com/1099-4300/21/7/715

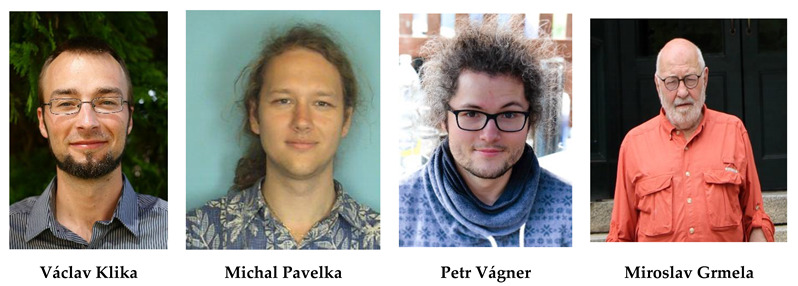



When proposing a model of real-world phenomena on any level of description, one has essentially two choices: (i) Direct constitutive modelling (i.e., proposing closures for all additional quantities, so that the final set of equations are well-posed; (ii) Reductions or extensions of known and well-established models on different levels of description.

In the former approach, one relies on constraints following from the second law, but when non-standard state variables are introduced, e.g., to reflect material structure or nonlocality, the guidance stemming from the second law constraints can be too weak. The latter approach addresses this issue by relating the state variables among different levels and hence guaranteeing both consistency and interpretability of the non-standard state variables as well as of phenomenological coefficients. Note that even the entropy itself has a clear meaning only as a potential relating to two levels of description.

There are multiple approaches to the transition among levels, some being more mathematical than others, e.g., homogenisation theory or geometric singular perturbation theory. We take a different route that focuses on the thermodynamical structure and properties of the models. This allows us to gain additional information and insights, although, admittedly, we cannot control or rigorously estimate errors in such approximations.

Our work offers an estimate of not only the reduced (also known as the upscaled) model but also the evolution driving the transition between the two levels using a method called Dynamic MaxEnt (DynMaxEnt). The method is based on the explicit recognition of the state and conjugate variables, which can relax towards the respective quasi-equilibria in different ways. Detailed state variables are reduced using the usual principle of maximum entropy (MaxEnt), whereas relaxation of the conjugate variables guarantees that the reduced equations are closed. Moreover, an infinite chain of consecutive DynMaxEnt approximations can be constructed. 

We illustrate this method on a particle with friction, complex fluids (equipped with conformation and Reynolds stress tensor), hyperbolic heat conduction, and magnetohydrodynamics.

**Topological Information Data Analysis** **[4]**

**Pierre Baudot, Monica Tapia, Daniel Bennequin and Jean-Marc Goaillard**

***Entropy*** **2019, *21*(9), 869;** https://www.mdpi.com/1099-4300/21/9/869

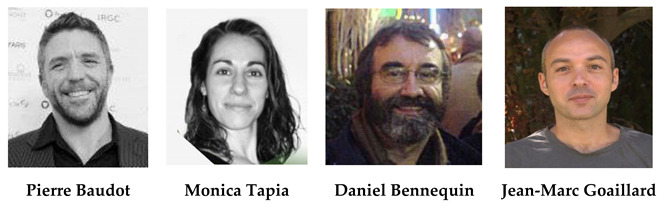



This paper presents methods that quantify the structure of statistical interactions within a given data set, and they were applied in a previous article. It establishes new results on the k-multivariate mutual information (*Ik*) inspired by the topological formulation of information introduced in a series of studies. In particular, we show that the vanishing of all *Ik* for 2 ≤ *k* ≤ *n* of n random variables is equivalent to their statistical independence. Pursuing the work of Hu Kuo Ting and Te Sun Han, we show that information functions provide coordinates for binary variables and that they are analytically independent of the probability simplex for any set of finite variables. The maximal positive *Ik* identifies the variables that co-vary the most in the population, whereas the minimal negative *Ik* identifies synergistic clusters and the variables that differentiate–segregate the most in the population. Finite data size effects and estimation biases severely constrain the effective computation of the information topology on data, and we provide simple statistical tests for the undersampling bias and the k-dependences. We give an example of the application of these methods to genetic expression and unsupervised cell-type classification. The methods unravel biologically relevant subtypes, with a sample size of 41 genes and with few errors. It establishes basic generic methods to quantify the epigenetic information storage and a unified epigenetic unsupervised learning formalism. We propose that higher-order statistical interactions and non-identically distributed variables are constitutive characteristics of biological systems that should be estimated in order to unravel their significant statistical structure and diversity. The topological information data analysis presented here allows for precisely estimating this higher-order structure characteristic of biological systems.

**Entropy Best Paper Award Committee**

Entropy Editorial Board.
